# Optical Coherence Tomography Angiography of Retinal Arterial Macroaneurysm before and after Treatment

**DOI:** 10.1155/2018/5474903

**Published:** 2018-03-06

**Authors:** Victoria S. Chang, Stephen G. Schwartz, Harry W. Flynn

**Affiliations:** Department of Ophthalmology, Bascom Palmer Eye Institute, University of Miami Miller School of Medicine, Miami, FL, USA

## Abstract

A case of retinal arterial macroaneurysm (RAM) is presented with multimodal imaging, including commercially available optical coherence tomography angiography (OCT-A). Following treatment with intravitreal bevacizumab, reduction of flow signal through the RAM is documented. OCT-A provides useful information for the diagnosis and management of at least some patients with RAM, without the need for traditional fluorescein angiography.

## 1. Introduction

Retinal arterial macroaneurysm (RAM) is an acquired dilatation of a retinal arteriole typically within the first three bifurcations that may occur with varying degrees of hemorrhage, edema, and exudation. Older women are predominantly affected, and there are strong associations with systemic hypertension and arteriosclerotic disease [1]. Although RAMs may involute spontaneously, treatment can be beneficial in the setting of associated macular edema, exudate, or neurosensory retinal detachment [2].

Spectral domain optical coherence tomography (SD-OCT) and fluorescein angiography are widely used in the diagnosis and management of retinal vascular diseases. More recently, OCT angiography (OCT-A), a noninvasive imaging modality that provides structural and functional (blood flow) information from different layers of the retina and choroid, has become available [3].

Using the commercially available Cirrus 5000 with AngioPlex™ (Zeiss, Jena, Germany), the OCT-A findings of RAM in a patient treated with off-label intravitreal bevacizumab (Avastin, Genentech, South San Francisco, CA) are described. A 6 × 6 mm slab was used for all images, and no subsequent image processing was performed.

## 2. Report of a Case

**Figure 1:**
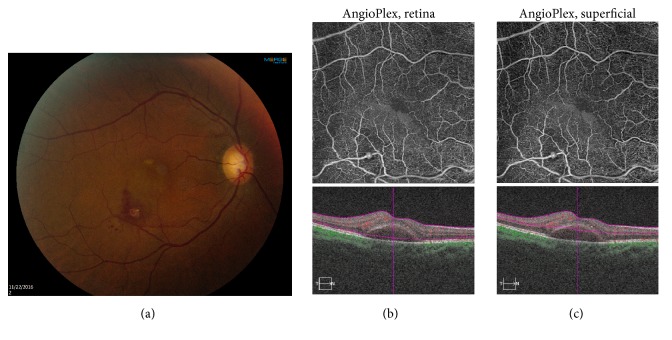
A 70-year-old female presented with decreased vision in the right eye. (a) Fundus photography shows the retinal arterial macroaneurysm (RAM) along the infratemporal vascular arcade, with surrounding subretinal hemorrhage and fluid. (b) TOP: optical coherence tomography angiography (OCT-A) retina slab clearly delineates the RAM. BOTTOM: spectral domain optical coherence tomography (SD-OCT) shows submacular fluid. (c) TOP: OCT-A superficial slab reveals the RAM. Bottom: SD-OCT shows submacular fluid.

A 70-year-old Haitian female with a history of chronic hypertension complained of decreased vision in the right eye. Visual acuity (VA) was 20/50. Fundus examination revealed a lesion along the infratemporal vascular arcade, consistent with RAM, and surrounding subretinal hemorrhage and fluid tracking into the macula (Figure 1(a)). SD-OCT showed submacular fluid (Figures 1(b) and 1(c), bottom). The RAM was well delineated on the OCT-A retina slab (Figure 1(b), top) and superficial slab (Figure 1(c), top). The patient was treated with intravitreal bevacizumab #1.

**Figure 2:**
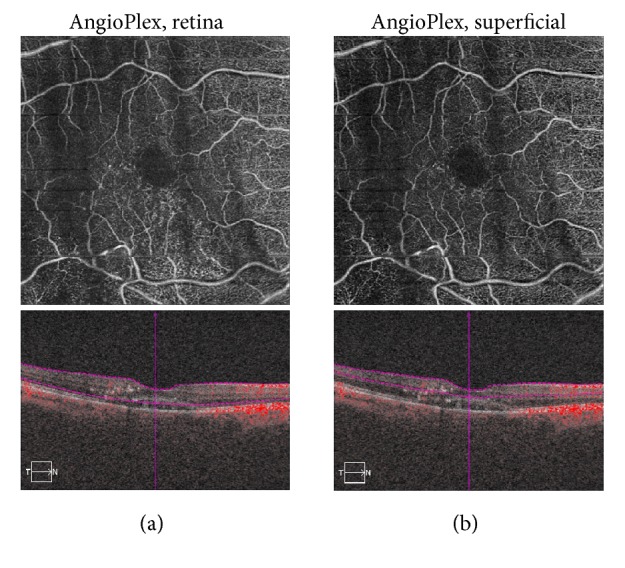
One month following presentation, following intravitreal bevacizumab #1. (a) TOP: optical coherence tomography angiography (OCT-A) retina slab demonstrates reduced signal due to artifact and decreased flow signal through the retinal arterial macroaneurysm (RAM). BOTTOM: spectral domain optical coherence tomography (SD-OCT) reveals reduced submacular fluid. (b) TOP: OCT-A superficial slab shows reduced signal due to artifact and decreased flow signal through the RAM. BOTTOM: SD-OCT reveals decreased submacular fluid.

One month later, the patient reported subjective improvement, although the VA remained 20/50. Fundus examination showed decreased blood and subretinal fluid, with lipid exudates in the macula. Fundus photography was not performed at this visit. SD-OCT showed reduced submacular fluid (Figures 2(a) and 2(b), bottom). Lipid exudates appeared as scattered hyperreflective signaling. The OCT-A retina slab (Figure 2(a), top) and superficial slab (Figure 2(b), top) demonstrated diminished signal due to artifact but also reduced flow signal through the RAM. The patient was treated with intravitreal bevacizumab #2.

**Figure 3:**
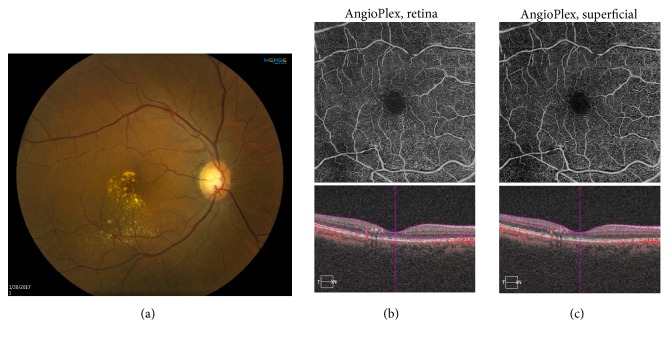
Two months following presentation, following intravitreal bevacizumab #2. (a) Fundus photography shows apparent sclerosis of the retinal arterial macroaneurysm (RAM) with lipid exudate in the macula. (b) TOP: optical coherence tomography angiography (OCT-A) retina slab demonstrates reduced flow signal through the retinal arterial macroaneurysm (RAM). BOTTOM: spectral domain optical coherence tomography (SD-OCT) shows restoration of the foveal contour. (c) TOP: OCT-A superficial slab reveals reduced flow signal through the RAM. BOTTOM: SD-OCT shows decreased submacular fluid.

Two months after presentation, VA improved to 20/30. Fundus examination showed apparent sclerosis of the RAM, with persistent lipid exudate in the macula (Figure 3(a)). SD-OCT showed further reduction of submacular fluid (Figures 3(b) and 3(c), bottom). The OCT-A retina slab (Figure 3(b), top) and superficial slab (Figure 3(c), bottom) demonstrated reduced signal flow through the RAM with persistent flow through the normal arteriole. The patient was observed.

**Figure 4:**
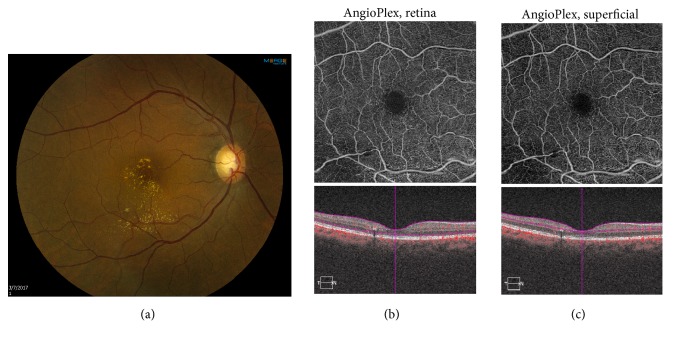
Four months following presentation. (a) Fundus photography demonstrates the sclerosed retinal arterial macroaneurysm (RAM). (b) TOP: optical coherence tomography angiography (OCT-A) retina slab reveals further reduced flow signal through the RAM. BOTTOM: spectral domain optical coherence tomography (SD-OCT) reveals resolution of the submacular fluid. (c) TOP: OCT-A superficial slab shows reduced flow signal through the RAM. BOTTOM: SD-OCT reveals improved macular contour.

Four months after presentation, VA improved to 20/25. Fundus examination showed persistent sclerosis of the RAM, with reduced lipid exudate in the macula (Figure 4(a)). SD-OCT showed restoration of the macular contour (Figures 4(b) and 4(c), bottom). The OCT-A retina slab (Figure 4(b), top) and superficial slab (Figure 4(c), bottom) showed further reduced signal flow through 4 the RAM with persistent flow through the normal arteriole. The patient was observed and was subsequently lost to follow-up.

## 3. Discussion

Many patients with RAM improve spontaneously, especially if the center of the macula is not involved. For patients with visual loss, treatment options include laser, antivascular endothelial growth factor (anti-VEGF) agents, and combination therapies [4]. Laser for RAM has been reported using yttrium aluminum garnet (YAG), conventional photocoagulation (argon or krypton), and subthreshold treatment using infrared diode [5]. If the RAM causes vitreous hemorrhage, then pars plana vitrectomy techniques may be considered [6].

In the present case, OCT-A demonstrated a focal outpouching of the vessel with a hyperreflective lumen consistent with an active RAM. The lesion became hyporeflective following anti-VEGF treatment, suggesting that it had involuted.

OCT-A can provide clinically useful information in the diagnosis and management of RAM. However, motion artifact and signal attenuation due to hemorrhage may cause degradation of the image quality. Nonetheless, OCT-A is able to produce high resolution images, while avoiding the long acquisition time and invasive nature of fluorescein angiography [7, 8].

In patients with RAM in whom an adequate OCT-A can be obtained, fluorescein angiography may not be necessary. Further experience with OCT-A may clarify the precise role of this technology in diagnosing and managing retinal vascular diseases.
